# TRPM2-mediated rise in mitochondrial Zn^2+^ promotes palmitate-induced mitochondrial fission and pancreatic *β*-cell death in rodents

**DOI:** 10.1038/cdd.2017.118

**Published:** 2017-07-28

**Authors:** Fangfang Li, Tim S Munsey, Asipu Sivaprasadarao

**Affiliations:** 1 Faculty of Biological Sciences, School of Biomedical Sciences, University of Leeds, Leeds LS2 9JT, UK; 2 Multidisciplinary Cardiovascular Research Centre, University of Leeds, Leeds LS2 9JT, UK

## Abstract

Rise in plasma free fatty acids (FFAs) represents a major risk factor for obesity-induced type 2 diabetes. Saturated FFAs cause a progressive decline in insulin secretion by promoting pancreatic *β*-cell death through increased production of reactive oxygen species (ROS). Recent studies have demonstrated that palmitate (a C_16_-FFA)-induced rise in ROS causes *β*-cell death by triggering mitochondrial fragmentation, but the underlying mechanisms are unclear. Using the INS1-832/13 *β*-cell line, here we demonstrate that palmitate generates the ROS required for mitochondrial fission by activating NOX (NADPH oxidase)-2. More importantly, we show that chemical inhibition, RNAi-mediated silencing and knockout of ROS-sensitive TRPM (transient receptor potential melastatin)-2 channels prevent palmitate-induced mitochondrial fission. Although TRPM2 activation affects the intracellular dynamics of Ca^2+^ and Zn^2+^, chelation of Zn^2+^ alone was sufficient to prevent mitochondrial fission. Consistent with the role of Zn^2+^, palmitate caused a rise in mitochondrial Zn^2+^, leading to Zn^2+^-dependent mitochondrial recruitment of Drp-1 (a protein that catalyses mitochondrial fission) and loss of mitochondrial membrane potential. In agreement with the previous reports, Ca^2+^ caused Drp-1 recruitment, but it failed to induce mitochondrial fission in the absence of Zn^2+^. These results indicate a novel role for Zn^2+^ in mitochondrial dynamics. Inhibition or knockout of TRPM2 channels in mouse islets and RNAi-mediated silencing of TRPM2 expression in human islets prevented FFA/cytokine-induced *β*-cell death, findings that are consistent with the role of abnormal mitochondrial fission in cell death. To conclude, our results reveal a novel, potentially druggable signalling pathway for FFA-induced *β*-cell death. The cascade involves NOX-2-dependent production of ROS, activation of TRPM2 channels, rise in mitochondrial Zn^2+^, Drp-1 recruitment and abnormal mitochondrial fission.

Obesity is a major risk factor for type 2 diabetes (T2D).^1, 2^ In most obese individuals, the enlarged adipose tissue releases abnormal amounts of free fatty acids (FFAs) into the blood stream. The resulting rise in plasma FFA levels leads to insulin resistance (impaired glucose uptake by muscle and adipose) and the consequent hyperglycaemia.^3^ Left unchecked, high levels of FFAs and plasma glucose lead to *β*-cell failure and apoptosis.^4, 5, 6, 7, 8, 9^ This process, termed as ‘glucolipotoxicity’, can lead to a permanent decline in *β*-cell mass, making the affected patients dependent on daily insulin injections. Unfortunately, none of the current therapies target the maintenance of *β*-cell mass to preserve endogenous insulin secretion. Improved understanding of the cellular and molecular basis for how glucolipotoxicity causes *β*-cell apoptosis is therefore important.

A key cellular target of glucolipotoxicity is mitochondria.^7, 9, 10, 11^ Studies have shown that exposure of *β*-cells to palmitate, one of the most abundant FFAs in the plasma, leads to extensive breakdown of healthy mitochondrial network to small, dysfunctional fragments, leading to mitochondrial dysfunction and *β*-cell death.^12^ Also in muscle cells, palmitate enhances mitochondrial fission, leading to their loss of insulin sensitivity.^13^ Consistent with these *in vitro* studies, *β*-cells from diabetes-prone obese Zucker rats show a 25% reduction in mitochondrial network,^14^ whereas C57BL/6 mice fed with high-fat diet (HFD) display fragmented mitochondria in their muscle.^13^ Furthermore, pharmacological inhibition of mitochondrial breakdown prevents HFD-induced T2D in mice.^13^ More importantly, patients with T2D and obesity show reduced mitochondrial size compared with lean subjects.^15, 16, 17^ Thus, multiple lines of evidence indicate a relationship between mitochondrial dynamics and obesity-associated T2D. However, our understanding of the cellular and molecular basis for how FFAs trigger abnormal mitochondrial dynamics is rudimentary.

In a normal cell, mitochondrial dynamics ensues that a healthy mitochondrial network is maintained through a careful balance between fission and fusion processes.^18, 19, 20, 21, 22^ Fission removes unhealthy parts of mitochondria, leaving the healthy portions of mitochondria to fuse and extend the network. Mitochondrial fission is catalysed by the dynamin-related protein, (Drp)-1, which constricts mitochondria at sites where specific adaptors (Mff, Mid49-51 and Fis1) are located.^18, 22^ Fusion is mediated by three GTPases: Mfn1, Mfn2, located in the outer mitochondrial membrane, and Opa1, present in the inner membrane.^18, 19, 20, 21, 22^

Nutrient stress tilts the balance in mitochondrial dynamics towards increased mitochondrial fission.^12, 13, 21^ Current evidence suggests that this shift is largely mediated by the reactive oxygen species (ROS) generated during the stress.^12, 13^ Furthermore, changes in intracellular Ca^2+^ dynamics regulate mitochondrial dynamics.^23, 24, 25, 26, 27^ Despite the tremendous progress, the mechanistic link between ROS production, Ca^2+^ dynamics and mitochondrial fission remains unclear.

Saturated FFAs are thought to induce *β*-cell apoptosis through a variety of mechanisms that include increased ceramide formation, oxidative stress and inflammation.^7^ In this study, we asked whether the oxidative stress-sensitive TRPM (transient receptor potential melastatin)-2 cation channel^28, 29, 30^ has a role in palmitate-induced mitochondrial fission in pancreatic *β*-cells. The underlying rationale was that palmitate-induced oxidative stress^31^ activates TRPM2 channels, leading to a raise in cytosolic Ca^2+^,^28, 29, 30^ and that the Ca^2+^, in turn, induces mitochondrial fragmentation. We have also examined the role of Zn^2+^ in mitochondrial fragmentation because activation of TRPM2 channels also affects intracellular Zn^2+^ dynamics.^32, 33^ Our results demonstrate that TRPM2 channels mediate palmitate-induced mitochondrial fragmentation in rodent *β*-cells by raising the mitochondrial levels of free Zn^2+^ and increasing Drp-1 recruitment to mitochondria.

## Results

### Palmitate-induced mitochondrial fragmentation is mediated by NOX2-induced ROS production

**Figure 1:**
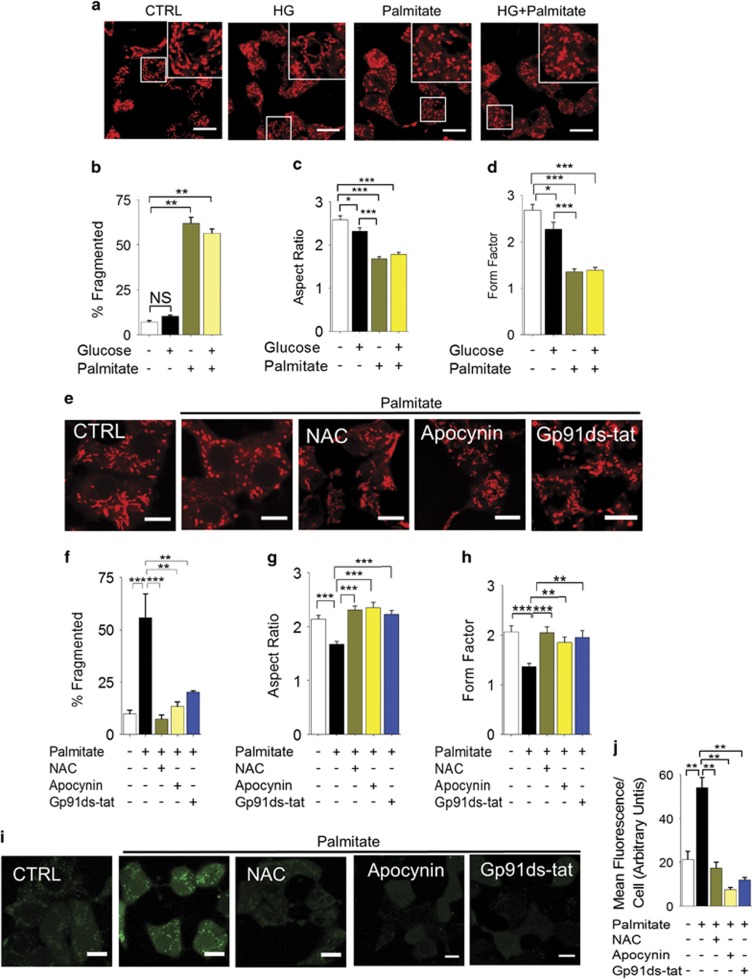
Palmitate causes mitochondrial fragmentation in INS1 832/13 cells through NOX2 activation. (**a–d**) Palmitate, but not high glucose, promotes mitochondrial fragmentation. (**a**) INS1-832/13 were incubated for 4 h in the medium alone (CTRL), or medium containing 20 mM glucose (HG), or 500 *μ*M palmitate, or HG plus 500 *μ*M palmitate. Confocal fluorescent images of cells stained with MitoTracker Red are shown; boxed regions are magnified in the top right corner of each image. (**b**) Mean±S.E.M. of percentage of cells displaying mitochondrial fragmentation; *n*=3. (**c** and **d**) Quantification of changes in mitochondrial morphology through estimation of aspect ratio (c) and form factor (d) of mitochondria following the treatments as in (**a**); *n*=3. (**e–h**) Palmitate-induced mitochondrial fragmentation is dependent on NOX2. (**e**) INS1-832/13 cells were exposed for 4 h to medium alone (CTRL), medium containing palmitate (500 *μ*M) with or without NAC (10 mM) or NOX2 inhibitors (20 *μ*M apocynin; 5 *μ*M gp91ds-tat). Representative images of cells stained with MitoTracker Red are shown. (**f**) Mean±S.E.M. of percentage of cells displaying mitochondrial fragmentation; *n*=3. (**g** and **h**) Changes in the aspect ratio and form factor of mitochondria following the treatments as in (**e**); *n*=3. (**i** and **j**) Palmitate increases ROS levels via NOX2 activation. Cells were treated as in **e**, and ROS were determined by staining the cells with DCF-DA. Representative images (**i**) and mean±S.E.M. (*n*=3) of fluorescence intensity due to ROS production (**j**) are shown. Scale bars in images: 10 *μ*m. In all bar charts, *P<0.05, **P<0.01, ***P<0.001, NS, not significant; one-way ANOVA with *post-hoc* Tukey’s test

We have examined the effect of palmitate (500 *μ*M, as a complex bovine serum albumin) and high glucose (HG: 20 mM) on mitochondrial dynamics of the rat pancreatic INS-1 832/13 *β*-cell line. We stained mitochondria with MitoTracker Red and examined the cells under a confocal microscope. The results showed long (>1.5 *μ*m), tubular, branched mitochondrial network in cells incubated in the standard RPMI medium containing 11.1 mM glucose. Raising the glucose concentration to 20 mM had little effect on mitochondrial morphology (Figures 1a–d). By contrast, consistent with a previous report,^12^ inclusion of palmitate in RPMI led to significant fragmentation of the network to small rounded fragments in the majority of cells that remained unchanged when the glucose concentration was raised to 20 mM (Figures 1a and b). Both the aspect ratio (length to width ratio) and the form factor (a measure of degree of branching) of mitochondria were significantly reduced (Figures 1c and d, and Supplementary Figure 1).

Previous studies have demonstrated that in *β*-cells palmitate induces ROS production by activating NADPH oxidase-2 (NOX-2).^34^ As ROS is a well-known trigger of mitochondrial fragmentation,^9, 12, 13, 18, 19, 20, 21^ we asked whether NOX-2 mediates palmitate-induced mitochondrial fragmentation. Consistent with this idea, inhibition of NOX-2 with the general NOX inhibitor apocyanin and the NOX2-specific peptide gp91ds-tat^35^ prevented palmitate-induced mitochondrial fragmentation, as effectively as the ROS scavenger N-acetyl-cysteine (Figures 1e–h). The ability of these reagents to fully suppress palmitate-induced ROS production (Figures 1i and j) indicate that NOX-2-derived ROS drive palmitate-induced mitochondrial fragmentation.

### TRPM2 channels mediate palmitate (via ROS)-induced mitochondrial fragmentation

**Figure 2:**
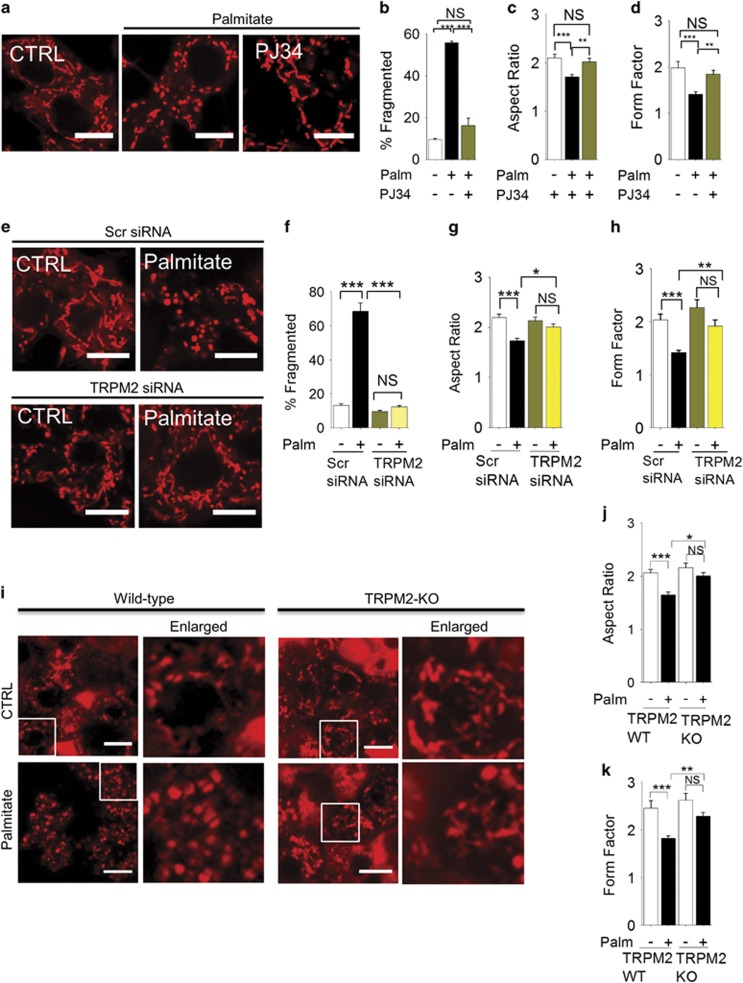
Inhibition of TRPM2 channels prevents palmitate-induced mitochondrial fragmentation. (**a–d**) Pharmacological inhibition of TRPM2 channels with PJ34 prevents palmitate-induced mitochondrial fragmentation. (**a**) Confocal images of INS1-832/13 cells exposed to medium alone (CTRL), or medium containing palmitate (500 *μ*M) with or without the PJ34 (10 *μ*M) for 4 h. Cells were stained with MitoTracker Red. (**b**) Mean±S.E.M. of percentage of cells displaying mitochondria fragmentation; *n*=3. (**c** and **d**) Changes in the aspect ratio (**c**) and form factor (**d**) of mitochondria following the treatments as in (**a**); *n*=3. (**e** and **h**) Silencing of TRPM2 RNA prevents palmitate-induced mitochondrial fragmentation. (**e**) INS1-832/13 cells were transfected with scr (scrambled) siRNA or TRPM2 siRNA for 60 h and then treated with palmitate as in **a**. Representative images of cells stained with MitoTracker Red are shown. (**f**) Mean±S.E.M. of percentage of cells displaying mitochondrial fragmentation; *n*=3. (**f–h**) Changes in aspect ratio (**g**) and form factor (**h**) of mitochondria following the treatments as in **e**; *n*=3. (**i**–**k**) Knockout of TRMP2 channels prevents palmitate-induced mitochondrial fragmentation in mouse pancreatic islet cells. (**i**) Islets were incubated in Optimem alone or Optimen containing 500 *μ*M palmitate for 5 days before staining with MitoTracker Red. Representative fluorescent images are shown, with the boxed regions enlarged. Islets isolated from each animal were subjected to two independent experiments; two animals were used for each group. (**j** and **k**) Changes in aspect ratio (**j**) and form factor (**k**) of mitochondria determined from cells randomly selected from islets stained as in **i**. Scale bars in images: 10 *μ*m. In all bar charts, *P<0.05, **P<0.01, ***P<0.001, NS, not significant; one-way ANOVA with *post-hoc* Tukey’s test

Ca^2+^ has a critical role in mitochondrial fission.^24, 26, 27^ Studies have shown that ROS can affect intracellular Ca^2+^ dynamics by targeting ion channels.^36^ We focussed our attention on TRPM2 calcium channels, because they are not only activated by ROS but their activation leads to *β*-cell death,^28, 29^ a process thought to precede excessive mitochondrial fission.^12, 21^ The results show that PJ34 (which inhibits TRPM2 channels by preventing the production of TRPM2 activator, ADP-ribose) inhibits palmitate-induced mitochondrial fragmentation (Figures 2a–d). In addition, TRPM2-siRNA, but not the control (scrambled) siRNA, inhibited palmitate-induced mitochondrial fragmentation (Figures 2e–h, and Supplementary Figure 2). Furthermore, palmitate caused mitochondrial fragmentation in islet cells of wild-type mice, but not TRPM2-knockout mice (Figures 2i–k). Finally, palmitate induced mitochondrial fragmentation in HEK293-TRPM2^tet^ cells^32^ only when they were induced to express TRPM2 channels (Supplementary Figure 3). Taken together, we conclude that TRPM2 channels mediate palmitate (via ROS)-induced mitochondrial fragmentation.

### TRPM2-mediated rise in mitochondrial Zn^2+^ is responsible for mitochondrial fragmentation

**Figure 3:**
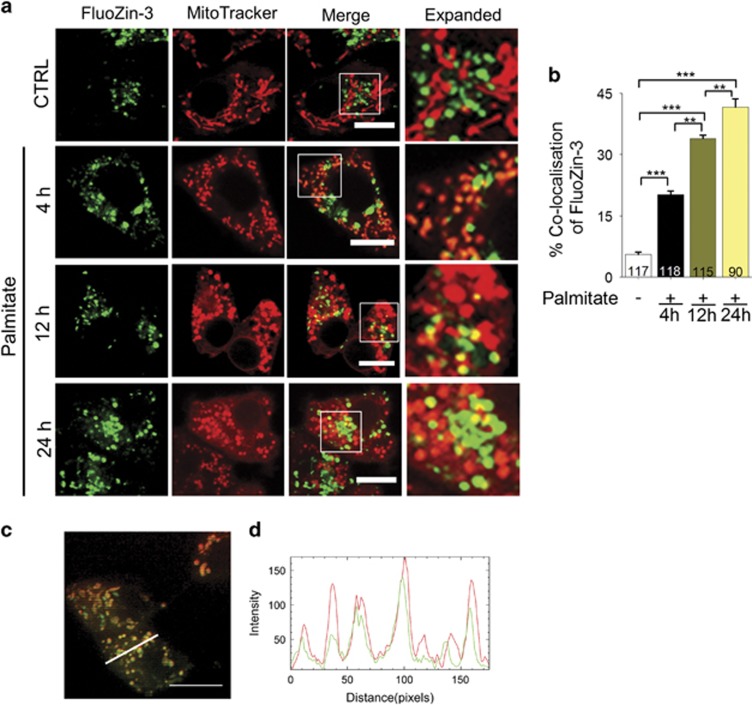
Palmitate causes a rise in Zn^2+^ levels of mitochondria. (**a**) Fluorescent images of INS1-832/13 cells co-stained for Zn^2+^ (FluoZin3-AM) and mitochondria (MitoTracker Red). Images were taken from cells exposed to medium alone (CTRL) or medium containing 500 *μ*M palmitate for 4, 12 or 24 h. Boxed regions in the merged images are magnified in the far right panels. Scale bars: 10 *μ*m. (**b**) Percentage co-localisation of FluoZin3-Zn^2+^ with MitoTracker Red was calculated from data in **a**; number of cells examined for co-localisation are shown within each bar. Data represent mean±S.E.M. (*n*=3); **P<0.01, ***P<0.001; one-way ANOVA with *post-hoc* Tukey’s test. (**c**) Fluorescent image of a *Z*-section of an INS1-832/13 cell after 4 h exposure to palmitate (500 *μ*M); the image was acquired using iSIM (for a movie of all *Z*-stacks see Supplementary material). (**d**) Plot of fluorescence intensity of FluoZin-3 stain (green) and MitoTracker Red (red) corresponding to the cross-section denoted with a white line in (**c**)

Studies have shown that TRPM2 activation not only increases cytosolic levels of Ca^2+^, but also of Zn^2+^.^32, 33, 37^ We have previously demonstrated that chelation of Zn^2+^ alone is sufficient to prevent TRPM2-mediated *β*-cell death.^32^ As excessive mitochondrial fission precedes cell death,^12, 21^ we speculated that changes in intracellular Zn^2+^ induce mitochondrial fragmentation. Accordingly, we examined the effect of palmitate on intracellular Zn^2+^ distribution by staining the cells with the Zn^2+^-specific dye, FluoZin-3-AM (green). We co-stained the cells with MitoTracker Red to correlate any changes in intracellular Zn^2+^ distribution with mitochondrial fragmentation (Figures 3a and b). As reported previously,^32^ in the untreated cells, FluoZin-3 stain was restricted to punctate structures. However, these structures did not show MitoTracker stain, indicating the absence of detectable levels of free Zn^2+^ in mitochondria. By contrast, within 4 h of palmitate treatment, mitochondria showed an unexpected rise in FluoZin-3 staining (yellow puncta representing co-localisation of FluoZin-3 stain with MitoTracker Red stain) (Figure 3a). Localisation of Zn^2+^ to mitochondria was obvious when we examined the Z-stack images of palmitate-treated cells (see Supplementary Movie) and plotted the intensity of Zn^2+^ and mitochondrial stain along a cross-section of the cell (Figures 3c and d). Prolonged palmitate treatment, however, led to mitochondrial membrane permeabilisation as indicated by the diffused MitoTracker Red stain in the cytoplasm.

**Figure 4:**
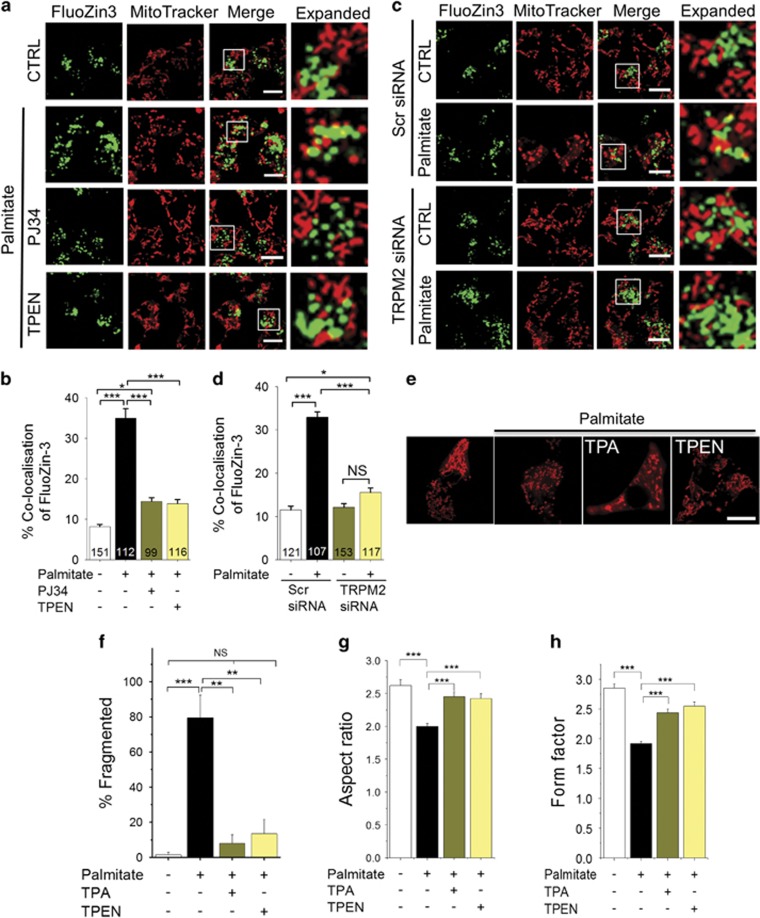
Inhibition of TRPM2 channels and chelation of Zn^2+^ prevents palmitate-induced Zn^2+^ rise in mitochondria and mitochondrial fission. (**a**) Fluorescent images of INS1-832/13 cells co-stained for Zn^2+^ (FluoZin3-AM) and mitochondria (MitoTracker Red). Images were taken after 12 h exposure to medium alone (CTRL) or medium containing 500 *μ*M palmitate minus or plus 10 *μ*M PJ34 or 0.5 *μ*M TPEN. Boxed regions in the merged images are magnified in the far right panels. (**b**) Percentage co-localisation of FluoZin-3 with MitoTracker Red was calculated from data in **a**; data represent mean±S.E.M. (*n*=3). (**c**) INS1-832/13 cells were transfected with scrambled siRNA or TRPM2 siRNA. 48 h after transfection, cells were treated with palmitate, stained and imaged as in **a**. Representative images are shown. (**d**) Percentage co-localisation of FluoZin-3 with MitoTracker calculated from data in **c**; data represent mean±S.E.M. (*n*=3). (**e**–**h**) Zn^2+^ chelation prevents palmitate-induced mitochondrial fragmentation. (**e**) Representative confocal images of INS1-832/13 cells exposed for 4 h to medium alone or medium containing 500 *μ*M palmitate in the absence or presence of TPA (10 *μ*M) and TPEN (1 *μ*M). (**f**) Mean±S.E.M. of percentage of cells displaying mitochondrial fragmentation following the treatments as in (e); *n*=3. (**g** and **h**) Changes in the aspect ratio (**g**) and form factor (**h**) of mitochondria determined from cells stained as in **e**; *n*=3. Scale bars in images: 10 *μ*m. In all bar charts, *P<0.05, **P<0.01, ***P<0.001, NS, not significant; one-way ANOVA with *post-hoc* Tukey’s test

We next examined the role of TRPM2 channels in palmitate-induced rise in mitochondrial Zn^2+^ and the impact of the Zn^2+^ on mitochondrial fragmentation. The results show that PJ34 (Figures 4a and b) and TRPM2-siRNA (Figures 4c and d) inhibited Zn^2+^ redistribution to mitochondria, as well as mitochondrial fragmentation. Similar results were obtained when H_2_O_2_ was used to induce oxidative stress (Supplementary Figure 4), with the exception that H_2_O_2_ treatment caused more global change in cellular Zn^2+^. Co-treatment of cells with the Zn^2+^ chelators, TPEN ((N,N,N′,N′-Tetrakis(2-pyridylmethyl)ethylenediamine)) and TPA (Tris(2-pyridylmethyl)amine), attenuated the ability of palmitate to induce mitochondrial fragmentation (Figures 4e–h). These results indicate that TRPM2-mediated increase in mitochondrial Zn^2+^ likely contributes to mitochondrial fragmentation.

### Palmitate-induced TRPM2 activation and Zn^2+^ redistribution cause loss of mitochondrial membrane potential

**Figure 5:**
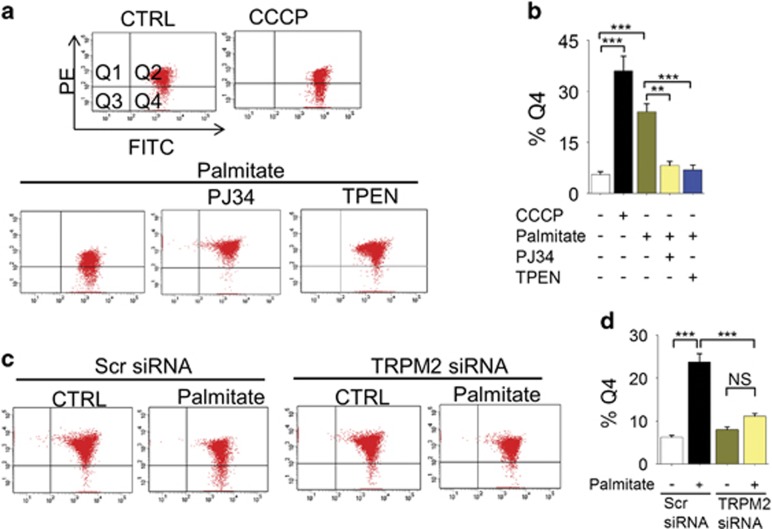
Inhibition of TRPM2 channels and chelation of Zn^2+^ rescues palmitate-induced loss of mitochondrial membrane potential (ΔΨmt). (**a**) INS1-832/13 cells were exposed to medium alone (CTRL), or medium containing 0.5 *μ*M protonophore CCCP (2 h), or 500 *μ*M palmitate minus or plus 10 *μ*M PJ34 or 0.5 *μ*M TPEN for 12 h. Cells were stained with JC-10 and assayed for loss of ΔΨm by flow cytometry. Q4 represents cells with depolarised Ψm. (**b**) Mean±S.E.M. of percent of cells with depolarised Ψmt; data from 3 independent experiments performed as in **a**. (**c**) INS1-832/13 cells were transfected with scrambled (scr) siRNA or TRPM2 siRNA; 60 h post-transfection, effect of palmitate on ΔΨmt was determined as in **a**. (**d**) Mean±S.E.M. of percent of cells with depolarised Ψmt; data from three independent experiments performed as in **c**. In **b** and **d**, **P<0.01, ***P<0.001; NS, not significant; one-way ANOVA with *post-hoc* Tukey’s test

Previous studies have shown that palmitate causes a loss of ΔΨmt in *β*-cells.^38^ We asked whether inhibition of mitochondrial fission mitigates palmitate-induced loss of ΔΨmt, because excessive mitochondrial fragmentation is associated with the loss of mitochondrial membrane potential (ΔΨmt).^18, 39, 40^ Consistent with the previous reports, palmitate caused a marked loss of ΔΨmt, as measured by the flow cytometry of JC-10 stained cells (the protonophore, carbonyl cyanide 3-chlorophenylhydrazone (CCCP) was used as a positive control). Inhibition of TRPM2 channels with PJ34, as well as chelation of Zn^2+^ with TPEN, prevented palmitate-induced loss of ΔΨmt (Figures 5a and b). Furthermore, TRPM2-siRNA, but not the scrambled siRNA, attenuated palmitate-induced loss of ΔΨmt (Figures 5c and d). Collectively, these data demonstrate that TRPM2-mediated changes in intracellular Zn^2+^ dynamics regulate palmitate-induced mitochondrial dysfunction.

### TRPM2-mediated changes in Zn^2+^ dynamics induce mitochondrial Drp1 recruitment

**Figure 6:**
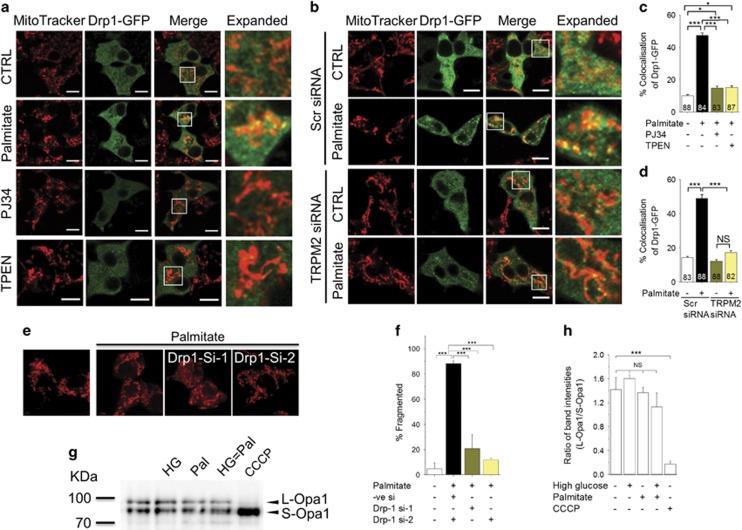
TRPM2 channels and Zn^2+^ mediates palmitate-induced Drp1 recruitment to mitochondria and subsequent mitochondrial fragmentation. (**a**) INS1-832/13 cells, transfected with Drp1-GFP, were exposed to medium alone (CTRL) or medium containing 500 *μ*M palmitate (4 h) minus or plus 10 *μ*M PJ34 or 0.5 *μ*M TPEN for 12 h. Cells were then stained with MitoTracker Red. Representative images are shown. (**b**) INS1-832/13 cells were co-transfected with Drp1-GFP and scrambled (scr) siRNA or TRPM2 siRNA; 60 h post-transfection, cells were treated with palmitate, stained and imaged as in **a**. Representative images are shown. (**c**,**d**) Mean±S.E.M. of percent co-localisation of Drp1-GFP with MitoTracker Red, calculated from three independent experiments performed as in (**a** and **b**), respectively; number of cells analysed are shown within bars. (**e** and **f)** Silencing of Drp1 prevents palmitate-induced mitochondrial fragmentation. (**e**) INS1-832/13 cells were co-transfected with p-mito-Cherry and siRNA against Drp1 (si-1 and si-2). Images show mito-Cherry-expressing cells following exposure to medium alone or medium containing palmitate (500 *μ*M). (**f**) Mean±S.E.M. of percentage of cells displaying mitochondrial fragmentation. (**g** and **h**) Opa-1 processing was not affected by high glucose and palmitate. (**g**) Western blotting of INS1-832/13 cells following various treatments as in Figure 1a. Positions of two marker proteins (Kda) and the two Opa-1 bands are indicated; CCCP (20 *μ*M) was used as a positive control. (**h**) Ratio of the L-Opa1 and S-Opa-1 band intensities, determined from three independent experiments performed as in (**g**). Scale bars in images: 10 *μ*m. In bar charts, numbers within each bar represent number of cells used for quantification; *P<0.05, ***P<0.001; NS, not significant; one-way ANOVA with *post-hoc* Tukey’s test

In a healthy cell, Drp-1, which catalyses mitochondrial fission, exists mostly in the cytoplasm, but in response to oxidative stress, it is translocated to mitochondria where it causes mitochondrial fission.^18, 19, 21^ Given that the TRPM2-mediated rise in mitochondrial Zn^2+^ causes mitochondrial fission (Figure 4), we next asked whether TRPM2 channels and Zn^2+^ induce Drp-1 recruitment. For this, we transfected INS1-832/13 cells with Drp-1-GFP (a construct designed to suppress endogenous Drp-1 expression^26^) and examined its localisation to MitoTracker Red-stained mitochondria. The results show that palmitate treatment increases the association of Drp-1-GFP with mitochondria (Figure 6a). Palmitate-induced Drp-1-GFP recruitment, as well as the associated increase in mitochondrial fragmentation, were inhibited by PJ34 (Figures 6a and c), TRPM2-siRNA (Figures 6b and d) and TPEN (Figures 6a and c). Consistent with the Drp-1 recruitment, RNA silencing of Drp-1 prevented palmitate-induced mitochondrial fission (Figures 6e and f).

Studies have shown that Opa1, when bound to the inner mitochondrial membrane (L-Opa1), catalyses mitochondrial fusion, but during stress it is cleaved to a soluble, short from (S-Opa1), which facilitates mitochondrial fission.^41^ However, western blotting data showed that palmitate, either alone, or in combination with high glucose, had no significant effect on the cleavage of L-Opa1 to S-Opa1. CCCP, as reported previously,^42^ caused complete cleavage of L-Opa1 to S-Opa1 (Figures 6g and h). Together, our results indicate that palmitate-induced mitochondrial fission is primarily mediated by TRPM2-dependent, Zn^2+^-mediated Drp-1-GFP recruitment to mitochondria.

### Comparison of the roles of Ca^2+^ and Zn^2+^ in mitochondrial fission

Previous studies have reported that Ca^2+^, by increasing the mitochondrial recruitment of Drp-1, stimulates mitochondrial fission.^24, 25, 26, 27^ However, our findings that chelation of Zn^2+^ alone was sufficient to prevent palmitate-induced Drp-1 recruitment (Figure 6) and mitochondrial fragmentation (Figure 4) raise doubts regarding the exclusive role of Ca^2+^ in mitochondrial fission. Unfortunately, none of the existing Ca^2+^ chelators, including the widely used BAPA-AM, which binds Zn^2+^ more avidly than Ca^2+^,^37, 43^ are suitable to distinguish the role of Ca^2+^ from that of Zn^2+^. To circumvent this, we delivered Ca^2+^ and Zn^2+^ to INS1-832/13 cells via ionophores and studied the individual effects of these ions. The specificity of these ionophores for Ca^2+^ and Zn^2+^ has been described previously.^37^

**Figure 7:**
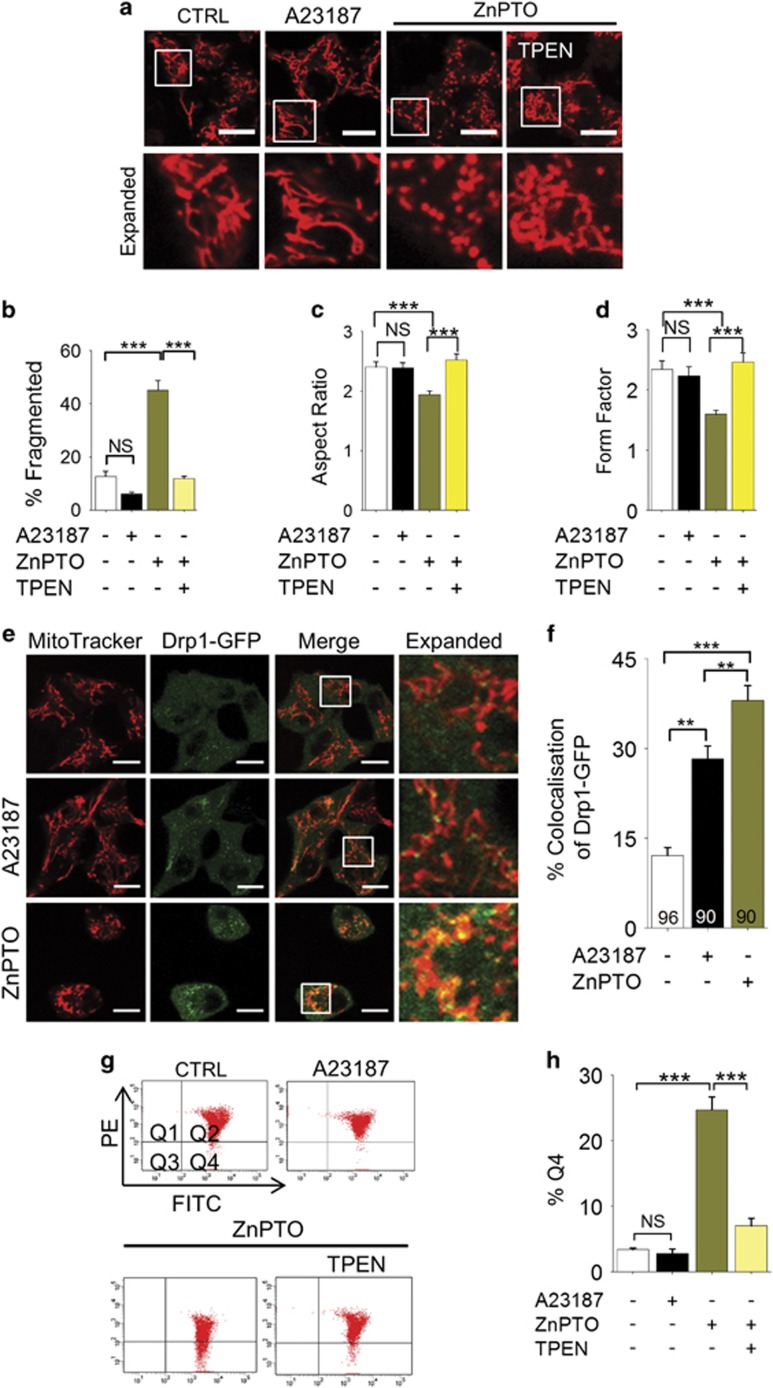
Zn^2+^, rather than Ca^2+^, has a dominant role in mitochondrial fragmentation. (**a**–**d**) Rise in intracellular Zn^2+^, but not Ca^2+^, leads to mitochondrial fission. (**a**) INS1-832/13 cells were treated with medium alone (CTRL), or medium containing A23187 (2 *μ*M) or ZnPTO (2 *μ*M) with or without TPEN (3 *μ*M) for 3 h followed by staining with MitoTracker Red. Representative images are shown. (**b**) Mean±S.E.M. of percentage of cells displaying mitochondria fragmentation. (**c**,**d**) Changes in the aspect ratio (**c**) and form factor (**d**) of mitochondria following the treatments as in **a**; *n*=3. (**e** and **f**) Rise in intracellular Zn^2+^ causes Drp1-GFP recruitment to mitochondria. (**e**) INS1-832/13 cells, transfected with Drp1-GFP, were treated with medium alone (CTRL) or medium containing A23187 (2 *μ*M) or ZnPTO (2 *μ*M) for 3 h, before staining for mitochondria; representative confocal images are shown. (**f**) Mean±S.E.M. of % co-localisation of Drp1-GFP with mitochondria calculated from three independent experiments performed as in (**e**); number of cells analysed are shown within bars. (**g** and **h**) Rising the intracellular Zn^2+^, but not Ca^2+^, causes loss of ΔΨm. (**g**) INS1-832/13 cells were treated as in (**a**), stained with JC-10 and sorted as in Figure 5a. (**h**) Mean±S.E.M. of percent of cells with mitochondrial depolarisation (Q4), calculated from three independent experiments performed as in (**g**). In all images, Scale bars: 10 *μ*m. In all bar charts, **P<0.01, ***P<0.001, NS, not significant; one-way ANOVA with *post-hoc* Tukey’s test

Rather unexpectedly, raising the cytosolic Ca^2+^ with A23187 failed to induce mitochondrial fragmentation (Figures 7a–d), although the ionophore caused significant recruitment of Drp-1-GFP to mitochondria (Figures 7e and f). Thus, although our data are consistent with the reported role of Ca^2+^ in Drp-1 recruitment,^24, 25, 26, 27^ they indicate that Ca^2+^-induced Drp-1 recruitment alone is not enough to induce mitochondrial fission. In contrast to A23187, raising the cytosolic Zn^2+^ levels with zinc-pyrithione (Zn-PTO) caused both Drp-1-GFP recruitment (Figures 7e and f) and mitochondrial fragmentation (Figures 7a–d). Consistent with the lack of effect on mitochondrial fission, A23187 failed to induce loss of ΔΨmt; by contrast, Zn-PTO caused a marked loss of ΔΨmt (Figures 7g and h). Together, these data support a key role that Zn^2+^ has in mitochondrial fission.

### Palmitate-induced *β*-cell apoptosis is mediated by TRPM2-mediated changes in Zn^2+^ dynamics

**Figure 8:**
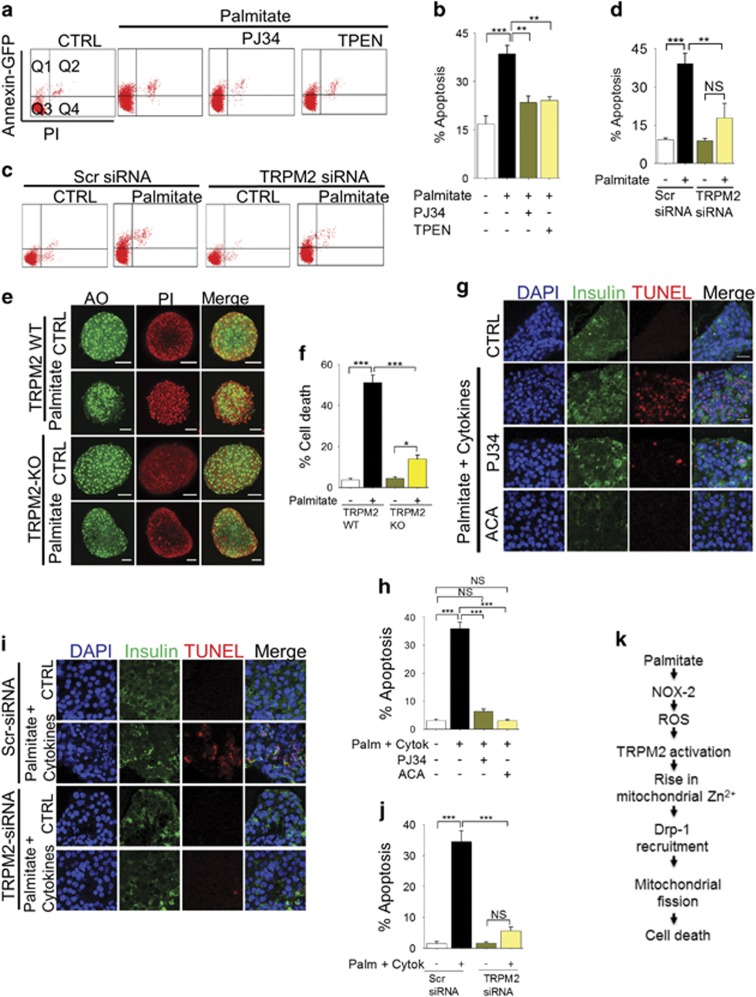
TRPM2 channels and Zn^2+^ mediate palmitate-induced apoptosis. (**a** and **b**) INS1-832/13 cells were treated with medium (CTRL) or medium containing 500 *μ*M palmitate minus or plus 10 *μ*M PJ34 or 0.5 *μ*M TPEN for 12 h. Cells were then stained with Annexin V-GFP and PI at 37 °C for 30 min and subjected to flow cytometry. Q1: live cells undergoing apoptosis; Q2: dead apoptotic cells; Q3: live cells; Q4: dead cells. Representative flow cytometry data (**a**) and mean±S.E.M. of percent apoptotic cells (Q1+Q2) from three independent experiments (**b**) are shown. (**c** and **d**) INS1-832/13 cells were transfected with scrambled siRNA or TRPM2 siRNA. Transfected cells were not treated (CTRL) or treated with 500 *μ*M palmitate for 12 h, before cell death assay as in **a**. Representative flow cytometry data (**c**) and mean±S.E.M. data for apoptosis from 3 independent experiments (**d**) are shown. (**e** and **f**) TRPM2 deficiency protects mouse islets from palmitate-induced apoptosis. (**e**) Mouse islets isolated from wild-type and TRPM2 KO mice were treated with Opti-MEM (CTRL) or Opti-MEM medium containing 500*μ*M palmitate for 5 days before staining with Acridine Orange (green:live) and PI (red:dead). (**f**) Mean±S.E.M. of percent cell death from three independent experiments performed as in (**e**). (**g** and **h**) TRPM2 inhibition prevents palmitate plus cytokine-induced human *β*-cell apoptosis. (**g**) Human islets were treated with medium alone (CTRL) or medium containing 500 *μ*M palmitate and cytokines (IFN-*γ*, 5ng/ml; IL-1*β*, 5ng/ml) with or without the indicated TRPM2 inhibitors (PJ34, 10 *μ*M, ACA, 15 *μ*M) for 7 days. The islets were then sectioned, and stained for insulin (immunostaining) and apoptotic cells (TUNEL); representative images are shown. (**h**) Mean±S.E.M. of percent apoptotic *β*-cell death in human islets; data are from experiments performed as in **g** on islets from three different donors. (**i** and **j**) Silencing of TRPM2 expression inhibits palmitate plus cytokine-induced human *β*-cell apoptosis. Human islets were transfected with scrambled (Scr) or TRPM2 siRNA and treated as in (**g** and **h**). (**i**) Representative images and (**j**) mean±S.E.M. data. In all bar charts, **P<0.01, ***P<0.001, NS, not significant; one-way ANOVA with *post-hoc* Tukey’s test. (**k**) Schematic of mechanism by which palmitate causes *β*-cell death, deduced form the data presented in this study

Given the above results, we examined the role of TRPM2 channels and Zn^2+^ in palmitate-induced *β*-cell apoptosis. Using flow cytometry of annexin-V-GFP and propidium iodide (PI)-stained cells, we first demonstrate that PJ34, TRPM2-siRNA (but not scrambled siRNA) and TPEN all inhibited palmitate-induced apoptosis of INS1-832/13 cells (Figures 8a–d). Furthermore, using Acridine Orange (green) and PI (red), we also demonstrate that palmitate triggers marked cell death in islets derived from wild-type, but not TRPM2 knockout mice (Figures 8e and f). These data demonstrate that palmitate induces *β*-cell death by activating TRPM2 channels.

We next examined the role of TRPM2 channels in human *β*-cell death. We found that, unlike murine islets, human islets were relatively resistant to palmitate-induced cell death when assayed with PI, although TUNEL staining showed some apoptosis (Supplementary Figure 5). We therefore combined palmitate with cytokines (IL-1*β* and IFN-*γ*), the levels of which also increase in obesity.^44^ The results show that this combination induces robust apoptosis of human *β*-cells, which was completely inhibited by TRPM2 inhibitors, PJ34 and ACA (N-(p-amylcinnamoyl)anthranilic acid)^28, 29^ (Figures 8g and h). Furthermore, transfection of human islets with TRPM2-siRNA, but not scrambled siRNA, prevented palmitate plus cytokine-induced apoptosis of *β*-cells (Figures 8i and j). These results support a role for TRPM2 channels in palmitate plus cytokine-induced human *β*-cell death.

## Discussion

Obesity is a major contributing factor to the fast growing epidemic of T2D.^1, 2^ In obesity, plasma levels of diabetogenic factors such as FFA and pro-inflammatory cytokines increase, leading to reduced insulin sensitivity and progressive decline in pancreatic *β*-cell mass.^3, 4, 5, 9^ Here we describe a novel signalling pathway by which palmitate, an experimental paradigm for FFA, causes *β*-cell death; the pathway includes the following sequence of events: (i) generation of ROS through NOX-2 activation, (ii) activation of TRPM2 channels by the resultant rise in ROS, (iii) rise in mitochondrial Zn^2+^, (iv) Zn^2+^-induced Drp-1 recruitment and mitochondrial fragmentation, and eventually, (v) *β*-cell death (Figure 8k). These results point towards new therapeutic opportunities for the treatment of obesity-induced T2D.

We investigated the mechanism by which palmitate induces abnormal mitochondrial fission.^12^ We first tested the possibility that this effect of palmitate could be related to its ability to increase ROS production, because ROS are potent stimulants of mitochondrial fission.^12, 18, 19, 20, 21^ *β*-Cells can generate ROS through a variety of mechanisms, including via activation of NOX,^34, 45^ of which NOX2 is important.^46^ We found that palmitate fails to induce mitochondrial fission in the presence of the NOX-2 specific peptide, gp91-ds-tat.^35^ (Figures 1e–h). These results indicate that NOX-2-derived ROS mediate palmitate-induced mitochondrial fission. Interestingly, previous studies have shown that Zucker diabetic rats generate ROS by activating NOX,^47^ and that deletion of the NOX-2 gene protects mice from high-fat diet induced T2D.^46^ Given the relationship between mitochondrial fission and T2D,^21^ our findings suggest that NOX-2-derived ROS might play a role in obesity-induced T2D by triggering mitochondrial fission.

Studies have shown that Ca^2+^ dynamics have an important role in ROS-induced mitochondrial fission;^24, 25, 26, 27^ however, the underlying Ca^2+^ handling mechanisms are not known. In this study, we found that pharmacological inhibition, siRNA-mediated knockdown and genetic knockout of TRPM2 channels prevent palmitate-induced mitochondrial fission in rodent models (Figure 2). This finding is indeed consistent with the properties of TRPM2 channels, namely, ability to be activated by ROS and conduct Ca^2+^ ions.^28, 29, 30^ However, TRPM2 channels not only permeate Ca^2+^ but also affect intracellular Zn^2+^ dynamics.^32, 33, 37^ In an attempt to exclude a role for Zn^2+^, we have used TPEN, which, at low concentrations, chelates Zn^2+^, but not Ca^2+^,^32, 37, 43^ and TPA. Unexpectedly, both the chelators blocked palmitate-induced mitochondrial fragmentation (Figures 4a and b). Direct delivery of Zn^2+^ via pyrithione (Zn-PTO) pheno-copied the effect of palmitate on mitochondria (Figures 7a–d), providing additional support to the role of Zn^2+^ in mitochondrial fragmentation. Together, these results indicate that TRPM2-mediated changes in intracellular Zn^2+^ dynamics have a key role in palmitate-induced mitochondrial fission.

To get a better insight into how Zn^2+^ might affect mitochondrial dynamics, we asked how palmitate affects intracellular Zn^2+^ dynamics. Intriguingly, we found palmitate to cause a rise in mitochondrial Zn^2+^ (Figure 3) and inhibition of TRPM2 channels prevented this rise (Figures 4a–d). These results suggested that TRPM2-mediated rise in mitochondrial Zn^2+^ may underlie palmitate-induced mitochondrial fission. Although little is known about how Zn^2+^ affects mitochondrial dynamics, studies have demonstrated that rise in mitochondrial Zn^2+^ causes a loss of ΔΨmt.^48^ Given the close association between ΔΨmt loss and abnormal mitochondrial fission,^18, 19, 21, 39^ we reasoned that inhibition of TRPM2-mediated rise in mitochondrial Zn^2+^ would prevent loss of ΔΨmt. Consistent with this reasoning, we found palmitate to cause a loss of ΔΨmt, which was prevented by TRPM2 inhibition (Figures 5a–d) and TPEN pre-treatment (Figures 5a and b). Taken together, we conclude that TRPM2-mediated rise in mitochondrial Zn^2+^ leads to mitochondrial fragmentation and the associated loss of ΔΨmt. Exactly how TRPM2 channels promote the rise in mitochondrial Zn^2+^, however, remains to be investigated.

To get a mechanistic insight into how TRPM2 signalling regulates mitochondrial fragmentation, we have examined Drp-1-GFP recruitment to mitochondria. Consistent with the previous report,^12^ we found palmitate to increase Drp-1-GFP recruitment to mitochondria (Figures 6a–c). This recruitment was inhibited by chemical inhibition of TRPM2 and TRPM2-siRNA (Figures 6a–d). Recent studies have reported that during stress the long (L) form of Opa1 is cleaved to a short (S) form and the short form promotes mitochondrial fission. However, we found palmitate to have no effect on conversion of L-Opa1 to S-Opa1, indicating a lack of role for Opa1 in palmitate-induced mitochondrial fission. Thus TRPM2 channels mediate palmitate-induced mitochondrial fission by promoting Drp-1 recruitment to mitochondria.

We next examined the role of Zn^2+^ in Drp-1-GFP recruitment. Our results demonstrate that chelation of Zn^2+^ with TPEN prevents palmitate-induced Drp-1-GFP recruitment to mitochondria. (Figures 6a and c). Furthermore, direct delivery of Zn^2+^ via Zn-PTO induced recruitment of Drp-1-GFP and mitochondrial fission (Figures 7a–f). These results support a novel role for Zn^2+^ in Drp-1-GFP recruitment and mitochondrial fission. However, there is compelling evidence in the literature that Ca^2+^ promotes Drp-1 recruitment to mitochondria.^24, 25, 26, 27^ As the available Ca^2+^ chelators bind Zn^2+^ more avidly than Ca^2+^,^37, 43^ we were unable to use chelators to distinguish Ca^2+^-induced Drp-1 recruitment from that induced by Zn^2+^. We have therefore used an alternative strategy where we raised cytosolic Ca^2+^ with A23187. Consistent with the previous reports, A23187 did increase Drp-1-GFP recruitment to mitochondria, but, unlike Zn-PTO, A23187 failed to induce mitochondrial fragmentation in INS1-832/13 cells under the conditions used (Figures 7e and f). The reason why, despite inducing Drp-1 recruitment, Ca^2+^ failed to induce mitochondrial fission is unclear, but emerging evidence indicates that Drp-1 recruitment alone is not sufficient to induce mitochondrial fission. For example, Friedman *et al.*^49^ reported that preconstruction of mitochondria by the ER tubules is essential for Drp-1 to affect mitochondrial fission. A more recent study has demonstrated that Drp-1 recruitment must be followed by dynamin-2 recruitment to cause mitochondrial fission.^50^ Although further studies are required to appreciate the role of Zn^2+^ in the complex set of events leading up to mitochondrial fission, the fact that Zn^2+^ causes mitochondrial fission indicates that it could play a role in ER-mediated pre-constriction or dynamin-2 recruitment. Precisely how TRPM2 activation affects the distribution of Ca^2+^ and Zn^2+^ between the various intracellular compartments and how the two ions co-ordinate mitochondrial fission remains to be investigated.

Prevention of lipo-toxicity can be viewed as a potential strategy to mitigate obesity-associated T2D, especially by preventing irreversible damage to *β*-cells. We have tested whether it is possible to target the mechanisms uncovered in this study to prevent palmitate effects on *β*-cells. Our results indeed demonstrated that TRPM2-siRNA and TPEN prevent palmitate-induced INS1-832/13 cell death (Figures 8a–d). Furthermore, islets from TRPM2 knock-out mice were remarkably resistant to palmitate-induced mitochondrial fragmentation (Figures 2i–k) and cell death (Figures 8e and f). Intriguingly, human *β*-cells were relatively resistant to palmitate-induced *β*-cell death. However, when palmitate was combined with cytokines (IFN-*γ* and IL-1*β*), common components of diabetic milieu,^44^ *β*-cell apoptosis has markedly increased, which was completely rescued by chemical inhibitors of TRPM2 (Figures 8g and h) and TRPM2-siRNA (Figures 8i and j). Taken together, our results indicate that inhibition of TRPM2 channels protects *β*-cells from death induced by diabetic conditions.

In conclusion, we report that palmitate-induced mitochondrial fission and the consequent *β*-cell death can be prevented by inhibiting TRPM2-mediated changes in intracellular Zn^2+^ dynamics. Prevention of mitochondrial fragmentation is considered as a useful strategy to prevent obesity-induced diabetes;^13, 21^ however, no suitable targets have yet been identified. Although Drp-1 is a potential target, deletion of Drp-1 gene was found to be embryonically lethal,^51^ making Drp-1 a high-risk drug target. The discovery of the signalling events (mediated by NOX-2, TRPM2 and Zn^2+^) upstream of Drp-1 thus promises new therapeutic opportunities for obesity-induced T2D. Consistent with this idea, previous studies have reported that knock-out of genes encoding NOX2 (Xiang *et al.*^46^) and TRPM2 channels,^32^ and administration of Zn^2+^ chelators,^52^ protect mice from oxidative stress induced *β*-cell death and hyperglycaemia.

## Materials and Methods

### Reagents

FluoZin-3, AM, MitoTracker Red, Acridine Orange, MitoSOX Red, H2DCF-DA and Lipofectamine 2000 were obtained from Thermo Fisher Scientific, Waltham, MA, USA. JC-10 was from ATT Bioquest.All other chemicals were obtained from Sigma-Aldrich. (St. Louis, MO, USA). SiRNA (5′-CUGUCUAAGAUCAAUUACAACCU-3′) against rat TRPM2 and human TRPM2 (5′-GAAAGAAUGCGUGUAUUUUGUAA-3′)^37^ were designed using siDirect and custom made by Dharmacon (GE Healthcare, Chicago, USA). siRNAs against Drp-1 (Drp-1-si1: 5′-ACGUCAGAUUAAGCGUCUATT-3′ catalog number SI01507107 and Drp-1-si2: 5′-GGUGGUCAGGAACCGACAATT-3′ catalog number SI01507114) were obtained from Qiagen (Hilden, Germany). Control siRNA was from Ambion (4390846). Drp1-GFP and Annexin V-GFP were kind gifts from Dr Stefan Strack (University of Iowa, Iowa City, IA, USA) and Professor Christoph Borner (Institute of Molecular Medicine and Cell Research, University of Freiburg, Freiburg, Germany) respectively. Gp91ds-tat was from AnaSpec, Inc. (Fremont, CA, USA). IL-1-*β* and IFN-*γ* were purchased from PEPROTECH (London, UK). *In Situ* Cell death Detection Kit (for TUNEL assay) was purchased from Sigma-Aldrich. Guinea pig polyclonal anti-insulin was from Dako (Ely, UK). Alexa-Fluor 488-conjugated anti-guinea pig IgG (1:1000, ab150185) was from Abcam (Cambridge, UK). DAPI-Fluoromount-G was purchased from SouthernBiotech (Cambridge Bioscience, UK). pmito-Cherry was constructed from pECFP-Mito (Clontech, Mountain View, CA, USA).

### Isolation of mouse islets and cell culture

Mouse islets were isolated from 8-10 week old wild-type (C57BL/6) or TRPM2 knockout mice as described previously.^32^ INS-1 832/13 cells and mouse islets were maintained in in RPMI 1640 -GlutaMAX-I (Gibco, Thermo Fisher Scientific, Waltham, MA, USA) medium supplemented with 10% heat-inactivated foetal calf serum, penicillin (100 U/ml), streptomycin (100 *μ*g/ml), 1 mM sodium pyruvate, 50 *μ*M 2-mercaptoethanol and 10 mM HEPES. Cultures were maintained at 37 °C under 5% CO_2_ and humidified atmosphere. HEK-293-TRPM2^tet^ cells, engineered to express TRPM2 channels when induced with tetracycline, were cultured and induced as described previously.^32^

### Treatments

For live-cell imaging, cells were grown in glass-bottomed 35 mm FluoroDish (World Precision Instruments, Hitchin, UK) dishes. Palmitate-BSA complex (stock) was prepared by mixing 100 mM palmitic acid (made up in ethanol) with 10% fatty acid-free bovine serum albumin (BSA) at 50 °C and stored at 4 °C. Stock solution of glucose (1 M) was made with Opti-MEM (Thermo Fisher Scientific, Waltham, MA, USA). Cells were transfected with siRNA (25 nM) and/ or plasmid DNA using Lipofectamine 2000 according to the instructions of the manufacturer (Thermo Fisher Scientific, Waltham, MA, USA).

### ROS measurement

ROS production was measured using the H_2_DCF-DA reagent. Following the various treatments, cells were incubated in HBSS (Gibco) containing 10 *μ*M H_2_DCF-DA for 30 min at 37 °C/ 5% CO_2_. After three washes with HBSS, cells were imaged using a LSM700 inverted confocal microscope fitted with a × 63x/1.4 NA oil objective (excitation, 488 nm; emission, 515-540 nm).

### Mitochondrial fragmentation

Following the desired treatments, cells were treated with 100 nM MitoTracker Red (10 min, 37 °C) in HBSS. After washing with HBSS, cells were imaged at 37 °C using a LSM700 inverted confocal microscope fitted with a × 63 oil objective; cells were excited at 548 nm and emissions captured at 562 nm. Changes in mitochondrial morphology (aspect ratio and form factor) were determined using NIH Image J 1.44p and the Mito-Morphology Macro as described.^53^ Aspect ratio and form factor were determined from 10 or more cells sampled form three independent experiments. A cell with more than 50% of its total mitochondria fragmented (length <0.8 *μ*m length) was scored as a cell with fragmented mitochondria. Results from three independent experiments were used for statistical analysis. Mitochondrial fission in islets was determined by staining the whole islets (5 islets for each condition) with MitoTracker Red (100 nM) for 5 min before washing with PBS. *Z*-sections of the islets were taken and stacked to generate *Z*-projected images.

### Live-cell imaging of mitochondria and intracellular Zn^2+^ distribution

To detect intracellular distribution of free Zn^2+^, cells were loaded with 1 *μ*M FluoZin3-AM in the presence of 0.02% Pluoronic-F127 (1 h, 37 °C) in HBSS. After washing with HBSS, cells were treated (10 min, 37 °C) with 100 nM MitoTracker Red to stain the mitochondria. Cells were washed with HBSS and imaged using a LSM700 inverted confocal microscope fitted with an × 63 oil objective at 37 °C and appropriate excitation (FluoZin3-AM, 488 nm; MitoTracker Red, 548 nm) and emission (FluoZin3-AM, 519 nm; MitoTracker Red, 562 nm) wavelengths. Some images were collected using iSIM (instant structured illumination microscope) fitted with an Olympus Water Immersion Objective × 60/1.2 NA Uplsapo 60xw, and 488 nm and 561 nm lasers.^54^

### Measurement of mitochondria membrane potential

Changes in mitochondrial membrane potential were monitored by staining the cells with JC-10, followed by cell sorting. Cells were grown in a 24-well plate to ~80% confluency. After the desired treatments, cells were detached with 300 *μ*l of trypsin-EDTA and pelleted by centrifugation (500 *g*, 5 min). After washing once with HBSS (500 *g*, 5 min), the pellet was resuspended in 500 *μ*l of JC-10 solution (5 *μ*M/HBSS) and incubated for 30 min at 37 °C/5% CO_2_. The resuspended cells were then loaded into LSRFortessa flow cytometer (BD Biosciences, Oxford, UK) and sorted using optimal settings for green (FL-1) and red (FL-2) fluorescence. Raw data were analysed with FACSDiva Software (BD Biosciences). Typically, the green (FITC) and red (PE-A) fluorescence values from 5000 cells were collected.

### Cell death assays

Cells were cultured in a 24-well plate to ~80% confluency. Following the desired treatments, both floating cells and attached cells were collected and centrifuged for 5 min at 500 *g*. After washing once with Annexin buffer (10 mM HEPES, 140 mM NaCl, 2.5 mM CaCl_2_, pH 7.4), pelleted cells were resuspended in 500 *μ*l of Annexin buffer containing Annexin V-GFP (40 ng/ml) and PI (10 *μ*g/ml) for 30 min in the dark. Samples were then analysed in LSRFortessa flow cytometer. Cytometer settings were optimised for green (FL-1) and red (FL-2) fluorescence, and the data were analysed with FACSDiva Software. Green (FITC) and red (PE-A) fluorescence values from 8000 cells were collected.

### Cell death in mouse islets

Islets from mouse pancreata were isolated as described previously.^32^ Briefly, pancreata were dissected out from mice, minced into pieces (~1 mm^3^) and digested with collagenase (Type IA; 1 mg/ml in HBSS) for 10 min with gentle agitation at 37 °C. After washing with RPMI 1640 medium, islets were hand-picked under a dissecting microscope and incubated overnight in RPMI 1640 medium before drug treatments. For toxicity assays, islets were incubated with freshly prepared palmitate (500 *μ*M):BSA (0.95%) complex with or without drugs in Opti-MEM for 5 days at 37 °C in a humidified atmosphere containing 5% CO_2_/95% air. Islets were stained with Acridine Orange (67 *μ*M) and PI (50 *μ*g/ml), and imaged using a LSM700 confocal microscope fitted with a × 63 oil objective at 37 °C, and appropriate excitation (Acridine Orange, 488 nm; PI, 548 nm) and emission (Acridine Orange, 519 nm; PI, 562 nm) wavelengths.

### TUNEL apoptosis assay of human islets

Human islets were purchased from Prodo Laboratories (Irvine, CA, USA). Islets were incubated in Opti-Mem without or with palmitate:BSA complex (500 *μ*M:0.95%) plus IL-1*β* (5 ng/ml) and IFN-*γ* (5 ng/ml) for 7 days at 37 °C in a humidified atmosphere containing 5% CO_2_/95% air. Treated islets were fixed with 4% paraformaldehyde overnight, frozen in dry ice and sectioned. Sections (5 *μ*m) mounted onto slides were washed once with PBS followed by 2 min permeabilisation (0.1% TritonX-100/ 0.1% sodium citrate/PBS) on ice. After washing with PBS, sections were incubated with the TUNEL reaction mix (50 *μ*l TUNEL reaction mix/450 *μ*l enzyme solution) containing anti-insulin antibody (guinea pig polyclonal anti-insulin, 1:500) for 1 h at 37 °C. After washing with PBS, sections were incubated for 1 h with Alexa488-conjugated secondary antibody (1:1000 in PBS). Sections were washed with PBS, treated with DAPI-Fluoromount-G overnight. Images were taken using a LSM700 inverted confocal microscope fitted with a × 63 oil objective, and appropriate excitation (Alexa488, 494 nm; Cy-3, 548 nm) and emission (Alexa488, 519 nm; Cy-3, 562 nm) wavelengths.

### Western blotting

Proteins in lysates of INS1-833/13 cells were separated on 10% SDS-PAGE and subjected to western blotting using mouse anti-OPA1 antibodies (1:2000 dilution, BD Transduction Laboratories, catalog number 612606, Oxford, UK) and HRP-conjugated goat ant-mouse IgG (1:5000 dilution, BioRad catalog number 172-1011, Oxford, UK). Bands were detected by chemiluminescence and quantified using Image J.

### Data analysis

Co-localisation between Zn^2+^ and mitochondria was analysed using the Imaris software (Bitplane). All experiments were performed at least three times (*n*) and the values presented as mean±S.E.M. Statistical significance was determined using the Student’s *t*-test or one-way ANOVA, followed by Tukey’s *post-hoc* test. Probability (*P*) values are indicated with *, ** and ***, which correspond to values of 0.05, 0.01 and 0.001, respectively.
