# Supplementary material for: TRPM2-mediated rise in mitochondrial Zn2+ promotes palmitate-induced mitochondrial fission and pancreatic β-cell death in rodents
Source: Cell Death Differ. 2017 Jul 28;24(12):1999–2012. doi: 10.1038/cdd.2017.118 (PMC5686341; doi:10.1038/cdd.2017.118)
Supplement: Supplementary Figures [file cdd2017118x1.pdf]

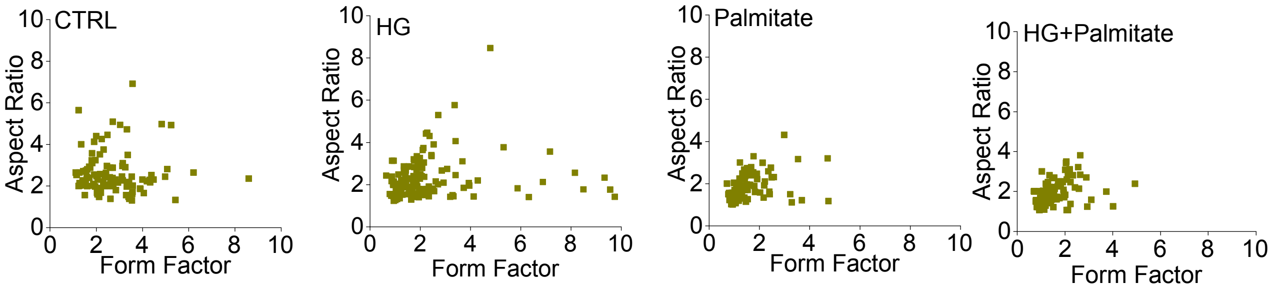

Supplementary Figure 1. Representative plots of form factor against aspect ratio of mitochondria from one image of a cell for each of the indicated conditions.

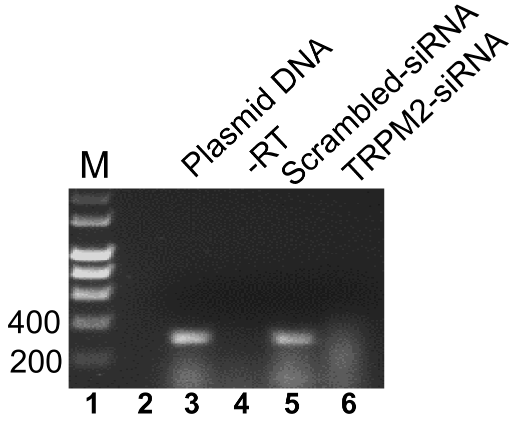

Supplementary Figure 2. Demonstration of silencing of TRPM2 mRNA expression by RNAi. Lane 1, HypperLadder I, Bioline; Lane 3, positive control; PCR product from TRPM2 plasmid DNA; Lanes 4-6, PCR products from mRNA isolated from INS1-832/13 cells (lane 4) and cells transfected with scrambled siRNA (lane 5) or TRPM2-siRNA (lanes 6). Lane 4 represents minus reverse transcriptase (-RT) control. The results show absence of TRPM2 band (lane 6) in TRPM2 siRNA transfected samples, but not scrambled siRNA controls (lane 5).

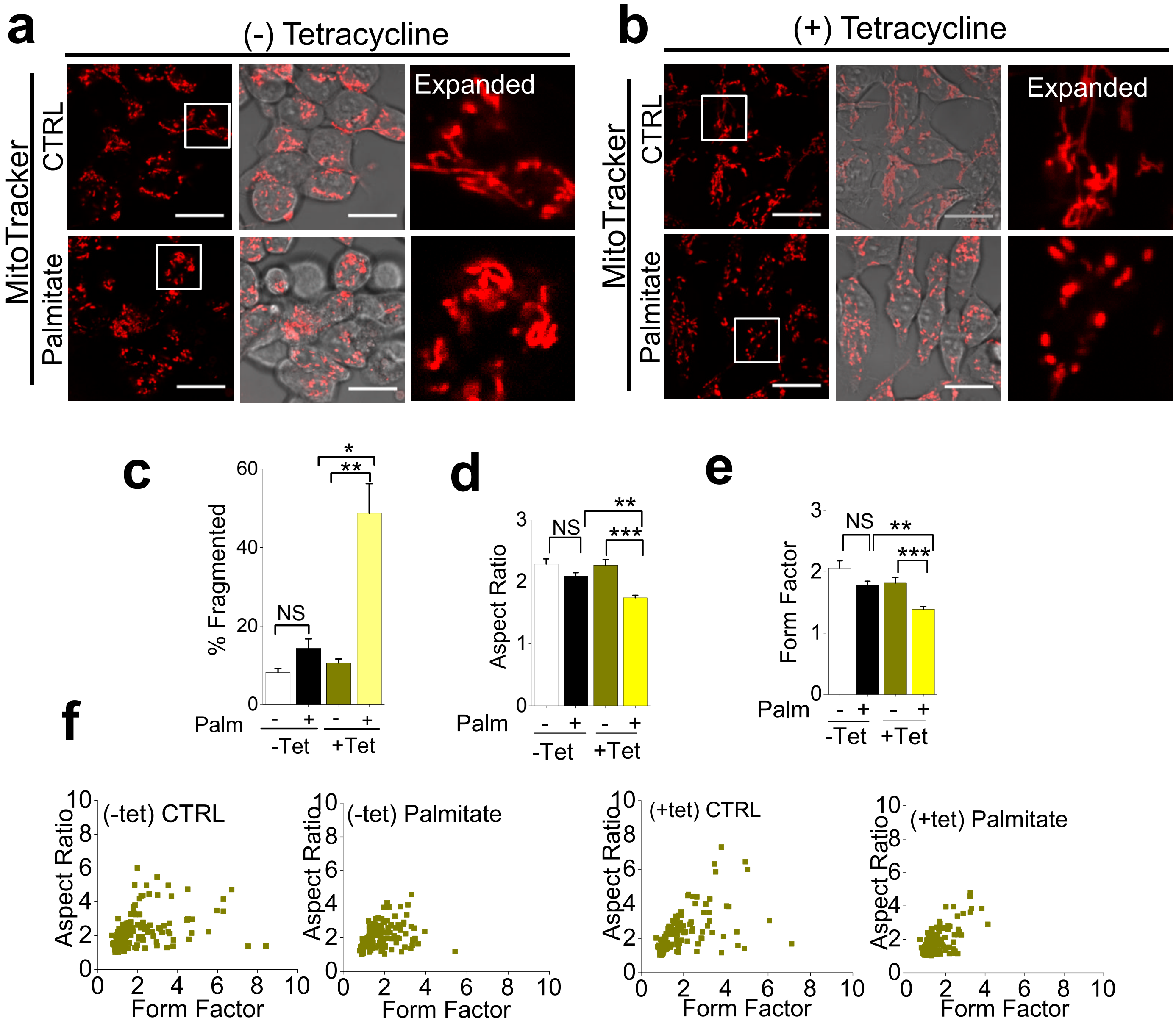

Supplementary Figure 3. Heterologous expression of TRPM2 channels enables palmitate to induce mitochondrial fragmentation in HEK-293 cells. (**a-b**) HEK-293-TRPM2^{tet} cells were either not treated or treated with tetracycline (1 µg/ml) to induce expression of TRPM2 channels. Cells were then treated with medium alone (CTRL) or medium containing 500 µM palmitate for 12 hrs ate 37°C. Cells were then stained with MitoTracker Red and imaged. Representative confocal images are shown. (**c-e**) show mean ± SEM data for percent cells showing fragmentation (c), and changes in the aspect ratio (d) and form factor (e) of mitochondria; n=3. Scale bars in images: 10 µm. In all bar charts, * $p < 0.05$, ** $p < 0.01$, *** $p < 0.01$, NS, not significant; one-way Anova with post-hoc Tukey test. (f) shows representative plots of form factor against aspect ratio of mitochondria from one image of a cell for each of the indicated conditions.

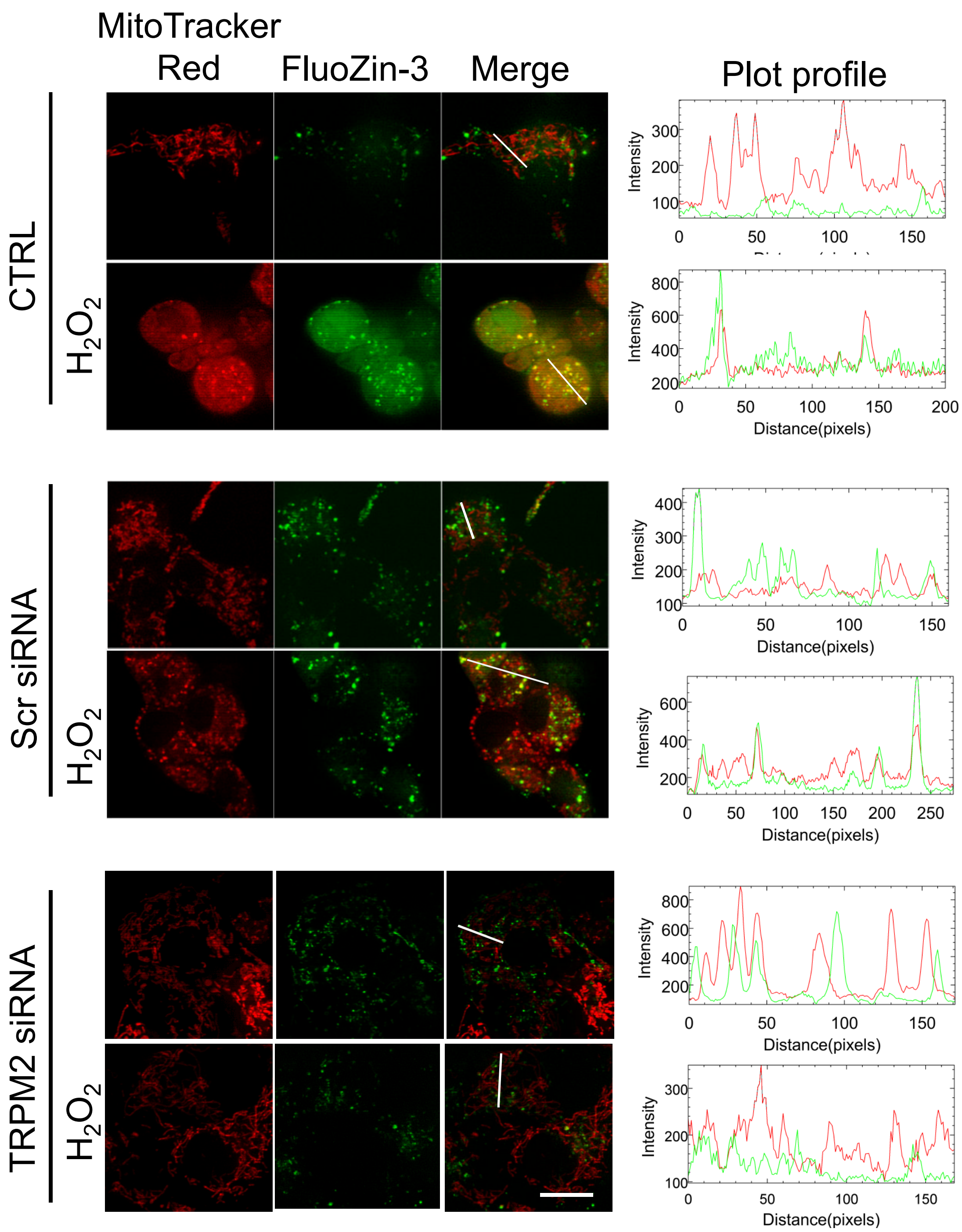

Supplementary Figure 4. Direct activation of TRPM2 channels with H_2O_2 leads to Zn^{2+} accumulation in mitochondria and mitochondrial fragmentation. INS1-832/13 cells were either not transfected (CTRL) or transfected with scrambled (Scr) siRNA or TRPM2 siRNA. Transfected cells were loaded with FluoZin-3-AM, exposed to medium alone or medium containing 50 μM H2O2 for 1 hr, before staining the mitochondria with MitoTracker Red. Representative FluoZin-3 (green) and MitoTracker Red (red) stained cells and the corresponding merged images are shown. On far right are shown pixel intensity of green and red puncta as a function of distance along the white line shown in merged images. The results show that TRPM2 siRNA, but not Scr siRNA, prevents H_2O_2-induced mitochondrial fragmentation and increased localisation of Zn^{2+} to mitochondria (as evident from yellow puncta in merged images and the plot profiles). Representative data from three independent experiments are shown.

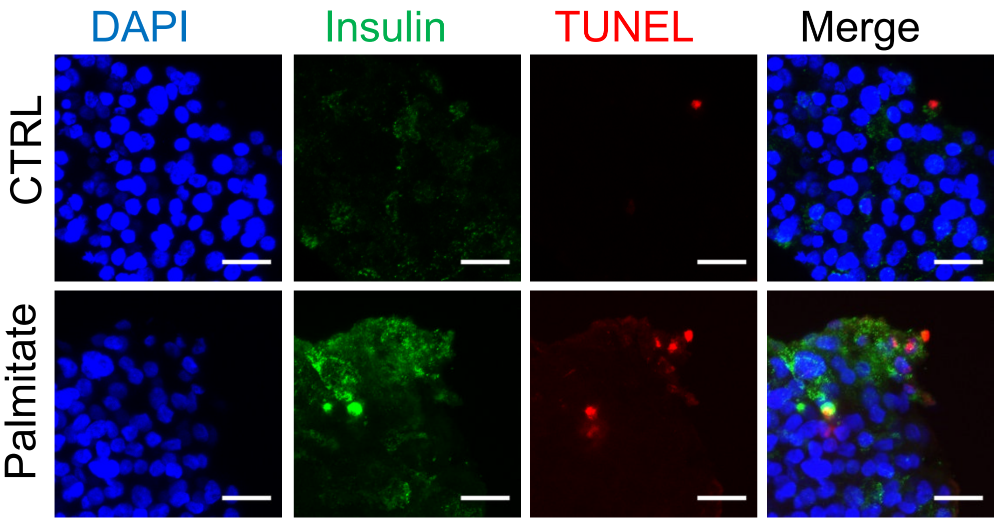

Supplementary Figure 5. Representative images of human islet sections showing palmitate-induced apoptosis. Human islets were exposed to Opti-Mem or Opti-Mem supplemented with palmitate:BSA complex (500 µM:0.95%) for 7 days at 37ºC in a humidified atmosphere containing 5% CO_2/95% air. Islets were fixed, sectioned, stained and imaged for Insulin and TUNEL stain as described in Materials and Methods section of the main text.
